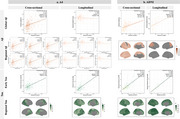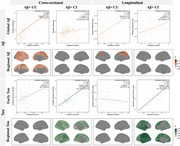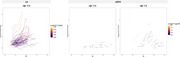# Plasma *p* ‐tau217 Predicting Concurrent Aβ and Longitudinal Tau Across the Brain

**DOI:** 10.1002/alz70861_109007

**Published:** 2025-12-23

**Authors:** Hasom Moon, Xi Chen

**Affiliations:** ^1^ Stony Brook University, Stony Brook, NY USA

## Abstract

**Background:**

Blood‐based plasma *p* ‐tau217 has been recognized as a promising biomarker with high sensitivity and accuracy for detecting Alzheimer’s disease (AD). However, its utility in inferring regional and longitudinal AD pathology is not fully understood. This study aimed to examine how plasma *p* ‐tau217 can reflect regional Aβ or tau burden and their longitudinal changes measured by PET.

**Method:**

We used data from the Anti‐Amyloid Treatment in Asymptomatic Alzheimer’s disease (A4) study (333 Aβ+ cognitively unimpaired older adults, age_mean_=72.2, females 57.1%) for primary analyses and Alzheimer’s Disease Neuroimaging Initiative (ADNI) (410 older adults including 203 Aβ+ older adults, age_mean_=75.1, females 49.3%) for validation. Baseline plasma *p* ‐tau217 was measured using Lilly (A4) and Fujirebio (ADNI) assays. Regional Aβ and tau were assessed by 18‐F Florbetapir Aβ PET and 18‐F Flortaucipir tau PET. Associations between *p* ‐tau217 and regional SUVRs were examined using regression adjusting for age, sex, and APOE ε4 carriership with Bonferroni correction.

**Result:**

In A4 consisting of Aβ+ cognitively unimpaired older people (Figure 1a), plasma *p* ‐tau217 was cross‐sectionally associated with Aβ across the brain and tau in temporo‐parietal regions. Longitudinally, *p* ‐tau217 predicted prospective tau accumulation in widespread cortical regions, but not Aβ change anywhere. These associations were replicated in Aβ+ participants in ADNI (Figure 1b). Interestingly, the associations between *p* ‐tau217 and cross‐sectional Aβ were driven by cognitively unimpaired individuals, but the cross‐sectional association and longitudinal prediction of *p* ‐tau217 for brain‐wide tau were only evident in cognitively impaired groups (Figure 2), likely due to limited tau change in cognitively unimpaired individuals in ADNI (Figure 3).

**Conclusion:**

Using two independent samples, we found strong evidence suggesting that blood‐based biomarker of AD can be used to infer brain‐wide neuropathology. High plasma *p* ‐tau217 reflects Aβ deposition across the brain in cognitively unimpaired older adults and predicts future brain‐wide tau accumulation across AD continuum. Plasma *p* ‐tau217, likely indexing the pre‐tangle form of tauopathy, may signal an earlier event prior to measurable PET change and can reflect Aβ plaques and the emergence of neurofibrillary tangles in the brain. Our findings highlight the great potential of plasma *p* ‐tau217 in early detection and disease monitoring of AD.